# Airborne cow allergen, ammonia and particulate matter at homes vary with distance to industrial scale dairy operations: an exposure assessment

**DOI:** 10.1186/1476-069X-10-72

**Published:** 2011-08-12

**Authors:** D'Ann L Williams, Patrick N Breysse, Meredith C McCormack, Gregory B Diette, Shawn McKenzie, Alison S Geyh

**Affiliations:** 1 Environmental Health Sciences, Johns Hopkins Bloomberg School of Public Health, 615 N. Wolfe Street, Baltimore, Maryland 21205, USA; 2 Pumonary and Critical Care Medicine, Johns Hopkins University School of Medicine, 1830 E. Monument Street, Baltimore, Maryland 21205, USA

## Abstract

**Background:**

Community exposures to environmental contaminants from industrial scale dairy operations are poorly understood. The purpose of this study was to evaluate the impact of dairy operations on nearby communities by assessing airborne contaminants (particulate matter, ammonia, and cow allergen, Bos d 2) associated with dairy operations inside and outside homes.

**Methods:**

The study was conducted in 40 homes in the Yakima Valley, Washington State where over 61 dairies operate.

**Results:**

A concentration gradient was observed showing that airborne contaminants are significantly greater at homes within one-quarter mile (0.4 km) of dairy facilities, outdoor Bos d 2, ammonia, and TD were 60, eight, and two times higher as compared to homes greater than three miles (4.8 km) away. In addition median indoor airborne Bos d 2 and ammonia concentrations were approximately 10 and two times higher in homes within one-quarter mile (0.4 km) compared to homes greater than three miles (4.8 km) away.

**Conclusions:**

These findings demonstrate that dairy operations increase community exposures to agents with known human health effects. This study also provides evidence that airborne biological contaminants (i.e. cow allergen) associated with airborne particulate matter are statistically elevated at distances up to three miles (4.8 km) from dairy operations.

## Background

The United States has witnessed the industrialization of the dairy industry over the last 40 years [1]. As a result, larger dairy facilities are now concentrated into fewer regions around the nation. The US Department of Agriculture (USDA) reports that between 1970 and 2000 the number of dairies nationwide decreased from 650,000 to 90,000. However, the number of dairy cows only declined from 12 to nine million while the average herd size increased 500% [1]. Though dairies are found in all 50 states, over a third of the all dairy animals are currently found in only two states [2]. For the purposes of this paper industrial scale dairies will be defined as operations that house over 500 animals.

Industrial food-animal production (IFAP) facilities are often located within or close to communities and reports of odors and concerns about health effects are common [3-5]. A number of airborne contaminants are produced by IFAP facilities, many which are unregulated. These include biological and biogenic aerosols, and gases such as ammonia, methane, and hydrogen sulfide. Unlike industrial sources, little is known about the airborne emissions from IFAP or potential community exposures. This is in part due to the virtual absence of agricultural air emission regulations and rural monitoring programs [6-9]. A Workgroup on Health Effects of Airborne Exposures from Industrial Scale Animal Operations concluded that there is a lack of data on community exposure to and health effects of odors and complex mixtures emanating from animal operations [10,11].

Within pre-existing communities in many areas of the country animal facilities have expanded both in size and processes in the last 10 to 15 years [2]. As a result, residents within these communities often found themselves suddenly living next to sprayfields where facility animal wastes are applied or barns containing thousands of animals. A few studies have suggested that the distance between a home and the facility may be an important determinant of exposure [12-16] however these studies did not specifically measure pollutant concentrations both inside and outside of homes nor did they evaluate pollutant concentrations at homes that were potentially unaffected.

**Figure 1:**
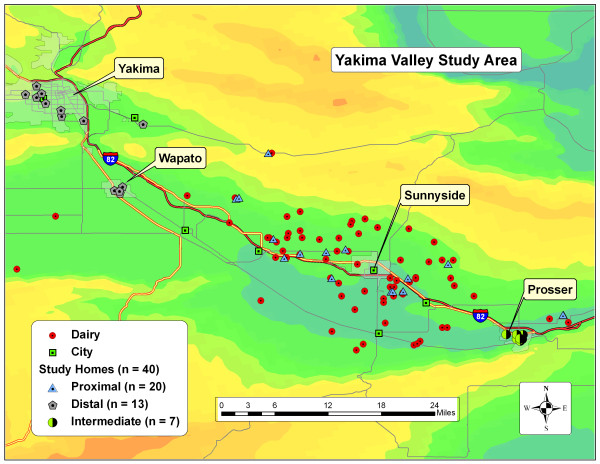
**Study area in Yakima Valley, Washington**. This figure geographically presents cities, dairies and study homes. The background color illustrates the elevation of the Central Yakima Valley. The lowest elevation of the valley follows Interstate 82 and is represented in blue-green. The valley is bounded to the north and south by ridges which run east-west and are shown by the color dark-yellow which represents the highest elevation. Elevations range from ~680-1580 ft above sea level.

To assess the impact of IFAP facilities on local community exposures to dairy-related contaminants, we conducted a study in Yakima Valley, Washington State where industrial dairy operations are concentrated in close proximity to surrounding communities. Dairy operations in the Yakima Valley are very large in terms of herd size and animal density. The 2009 Lower Yakima Valley Groundwater Quality Report identified 61 dairies between Prosser and the City of Yakima (49 mi, 79 km) housing approximately 207,000 cows [17] (Figure 1). While the 2007 USDA Agricultural Census reported that only 5% of all dairy operations have 500 cows or more, 72% of the operations in the Yakima Valley housed over 500 cows [2,18].

Agricultural dusts and crustal components typically make up the majority of airborne particles in farming communities. When airborne particulate matter (PM) settles, it can become resuspended by human activity, erosion and wind. It can remain airborne for weeks, and be transported for hundreds of miles [19,20]. Airborne particles from industrial scale animal operations can act as vectors for transmission of adsorbed chemicals, endotoxin, allergens and other biological agents [21-24].

Cows are the only source of Bos d 2, thus making it a specific indicator of dairy facilities in homes without resident cows. Bos d 2, a member of the family of lipocalins, allergic proteins, is associated with cow dander, sweat and urine. Cow allergen has been found at elevated concentrations in the air and dust inside barns, sheds, stables and the living quarters of dairy workers [25,26].

Ammonia is a gaseous contaminant resulting from the breakdown of manure and urine. It has a low odor threshold and is one of the primary factors in the diminishment of quality of life for residents of communities impacted by IFAP facilities [4,27]. It is corrosive and can be a powerful irritant to skin, eyes, and digestive and respiratory tissues [28].

In this paper we compare the distribution of dairy-related air pollutants (particulate matter and ammonia) and an allergen (Bos d 2 cow allergen) in homes close to dairy facilities compared to homes that are farther away.

## Methods

### Study Population

The study was approved by the Johns Hopkins Bloomberg School of Public Health and the Fred Hutchinson Cancer Research Center Institutional Review Boards. To be eligible for the study the consenting adult must have lived in the home for a minimum of six months. Exclusion criteria included homes that contained a resident dairy facility worker, cows on the premises, or individuals who worked in orchards or vineyards where manure spreading occurred. Homes that allowed smoking of any kind were also excluded as smoking influences PM concentrations.

Target recruitment included 20 homes defined as proximal and 20 homes defined as distal to a dairy operation. Proximal homes were defined as those within a ¼ mile (0.4 km) of an active dairy facility or adjacent sprayfield where dairy operation waste is applied. Distal homes were defined as those three miles (4.8 km) or more from an active dairy facility or sprayfield. Geographic areas of interest were identified in a two-stage approach using data available in ArcView GIS 9.2 (Redlands, CA). Georeferenced buffers were constructed which incorporated dairy facility and sprayfield location using data accessed through publicly available state databases [18,29]. Buffers were layered onto a parcel basemap from the Yakima County Government [30]. Associated parcel information was extracted from the Yakima County Tax Assessors [31] database. A total of 850 eligible proximal parcels with homes and more than 10,000 potentially eligible distal parcels with homes were identified.

### Environmental Monitoring

Environmental monitoring focused on airborne pollutants including PM, cow allergen (Bos d 2) and ammonia. While ammonia is not specific to cow waste, in this study there were no known ambient sources other than the animal facilities that would influence ammonia concentrations. In each home, matched indoor and outdoor samples were collected over a period of five days from June 10 to August 19, 2008. For each sampling event one proximal home and one distal home were paired and sampled on the same days.

Each air sampling set-up included a total dust (TD), ammonia (NH_3_), and a second hand smoke (SHS) sampler. The indoor set-up was placed in a common living area approximately 1.5 m off of the floor. The outdoor set-up was placed on a table or elevated surface that was approximately 1.5 m off the ground. Outdoor set-ups were protected by a weather resistant housing.

Airborne TD samples were collected using a closed-face VWR 37 mm sampling cassettes (VWR, Bridgeport, CT) pre-loaded with 37 mm Teflo^® ^filters, (Pall-Gelman, Ann Arbor, MI) at the Johns Hopkins Bloomberg School of Public Health (JHSPH). BGI sampling pumps (BGI, Waltham, MA) were pre- and post-calibrated using a Bios DryCal primary standard (Bios International, Butler, NJ). After sampling, filters were unloaded in a clean environment and stored in sealed petri-dishes at -20°C and then shipped overnight to JHSPH at 4°C and then stored at 4°C until analysis. Airborne TD samples were collected in order to collect a wide particle size range since the particle size associated with biogenic materials (e.g., cow allergen) is not known. Airborne TD mass concentration was determined by gravimetric analysis at JHSPH. Filters were pre- and post-weighed in a temperature and humidity controlled weighing room using a Mettler-Toledo MT5 microbalance (Mettler-Toledo, Inc., Columbus, OH) following EPA standard protocol, 40CFR50 Appendix L [32].

Airborne Bos d 2 concentrations were determined from the TD samples and analyzed by Indoor Biotechnologies, Inc. Charlottesville, VA. An ELISA assay, which had been modified based on previous immunoassay protocols to test for Bos d 2, was used for this analysis [33].

To confirm the nonsmoking status of the house, airborne nicotine concentrations were assessed using SHS monitors constructed at JHSPH. After sampling, monitors were stored at -20°C, shipped overnight to JHSPH at 4°C and then stored at 4°C until analyzed. Analysis for nicotine was conducted by gas chromatography with nitrogen-phosphorus detection as previously reported [34].

NH_3 _concentrations were measured using the Gradko passive NH_3 _sampler (Gradko International LTD, UK). The Gradko sampler has been validated for measurements of NH_3 _concentrations ranging from > 2.5 to 1000 μg/m^3^ (3.6 to 1436 ppb) [35,36]. After sampling, samplers were stored in a -20°C, shipped overnight to JHSPH at 4°C, then stored at -20°C until analyzed. Ammonia analysis was conducted by ion chromatography (Model 600 × IC, Dionex Corp. Sunnyvale CA) following the protocol described by Dionex [37].

All samples were submitted for laboratory analysis with proximity identifiers removed. Sample concentrations were blank corrected and duplicate samples were averaged and reported as one value. Values that were below the LOD were reported as 1/2 the LOD value [38].

### Home Characteristics

Housing characteristics including house age, number of people living in the home, dog and/or cat living in the house, presence of livestock, and presence of air conditioning were collected for each home by survey.

### Statistical Analysis

Exploratory data analysis was conducted using Microsoft Excel (Redmond, WA) and Stata SE11.0 (College Station, TX). Data were examined and descriptive statistics were generated to determine measures of central tendency and data distributions. Since environmental data are typically log-normally distributed, the Shapiro-Wilk test was used to determine normality to assess the appropriateness of the Student's t-test as a statistical method. The data were compared by group using the non-parametric Kruskal-Wallis test with a *p*-value threshold value of 0.05.

## Results

### Study Homes and Housing Characteristics

A convenience sample of 40 homes was recruited. Informed consent was obtained from the adult resident of the home who was to be the primary study contact. Of the 40 homes, 20 were designated proximal and 20 were designated distal. After the field study was concluded, additional ground truthing was conducted to reconfirm categorical assignment using satellite images. Distances were measured from dairy operations and adjacent sprayfields to study homes using the Google Earth "distance" tool. Seven of the homes originally categorized as "distal" homes were found to be within three miles (4.8 km) from dairy facility adjacent sprayfields. These homes were re-categorized as "intermediate" homes since they fell between the ¼ and three mile (0.4 km and 4.8 km) distance criteria. The reassignment of the intermediate homes created a total of 20 proximal, seven intermediate, and 13 distal homes (Figure 1). Analysis was conducted on these three groups.

Housing characteristics are summarized in Table 1. Overall, homes contained between three and four residents and had a mean housing age of 57 years. Distal and intermediate homes tended to be older than proximal homes with mean ages of 64, 79 and 45 years, respectively; only the difference between intermediate and proximal homes was statistically significant. Home characteristics based on number of people living in the home, the presence of air conditioning and pet ownership did not differ significantly by proximity.

**Table 1:** Housing Characteristics of Study Homes

Characteristics	Total (N = 40) mean ± SD (range)	Proximal (N = 20) mean ± SD (range)	Distal (N = 13) mean ± SD (range)	Intermediate (N = 7) mean ± SD (range)
Distance to facility (miles)*	3.42 ± 3.99 (0.4 - 11.5)	0.17 ± 0.61 (0.04 - 0.3)	8.65 ± 2.1 (5 - 12)	3.01 ± 0.28 (2.45 - 3.4)

# of people living in house	3.7 ± 1.88 (1 - 8)	3.3 ± 1.6 (1 - 6)	4 ± 2.2 (1 - 8)	4 ± 2.2 (2 - 8)

Age of house (years)	57 ± 31 (3 - 109)	45 ± 46 (5 - 107)	64 ± (3 - 99)	79 ± 22 (57 - 109)

	Total (N = 40) n (%)	Proximal (N = 20) n (%)	Distal (N = 13) n (%)	Intermediate (N = 7) n (%)

Dog (outside house)	27 (68)	14 (70)	8 (63)	4 (57)

Dog (inside house)	18 (45)	9 (45)	7 (54)	1 (14)

Cat (outside house)	12 (30)	8 (38)	3 (19)	3 (43)

Cat (inside house)	8 (20)	5 (25)	2 (13)	3 (43)

Other (chicken, horse, goat)	6 (15)	5 (25)	0 (0)	1 (14)

Live adjacent to sprayfield	15 (38)	15 (75)	0 (0)	0 (0)

Any air conditioning	28 (70)	11 (55)	10 (77)	7 (100)

*measured using Google Earth.

### Airborne Sample Results and Comparison of Study Homes

A summary of sampling results is presented in Table 2. TD concentrations ranged from two to 385 μg/m^3 ^(median: 22 μg/m^3^). Approximately 16% of airborne Bos d 2 samples were below detection with concentrations ranging from < 0.2 to 1.9 μg/m^3 ^(median: 0.4 μg/m^3^). Only nine percent of ammonia samples were below the limit of detection with results ranging from < 0.9 to 56 ppb (median: 6.0 ppb).

**Table 2:** In Home Airborne Sample Concentrations

Sample Type	samples (n)	% < LOD	LOD	mean	SD	min	median	max
**Bos d 2 μg/m^3^**	70	16	0.02	0.3	0.4	< 0.02	0.4	1.9

**NH_3 _ppb**	79	9	0.9	8.8	10.6	< 0.9	6.0	56.0

**TD μg/m^3^**	75	0	1.1	35.0	54.7	2	22	385

### Outdoor Air

**Figure 2:**
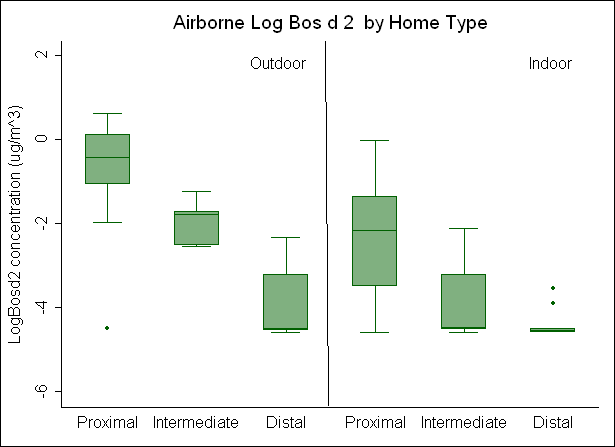
**Comparison of outdoor and indoor airborne concentrations of Bos d 2 between proximal, intermediate and distal homes**.

**Figure 3:**
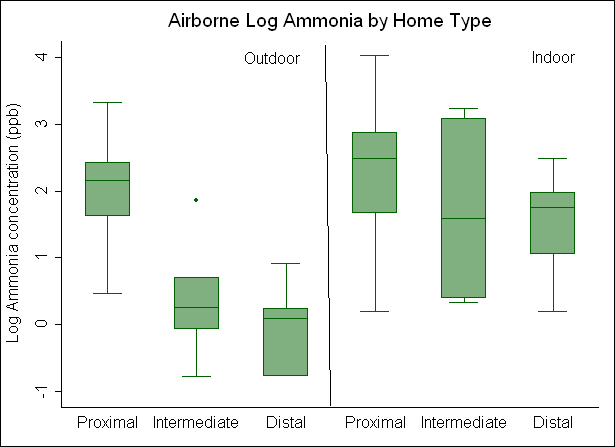
**Comparison of outdoor and indoor airborne concentrations of ammonia between proximal, intermediate and distal homes**.

**Figure 4:**
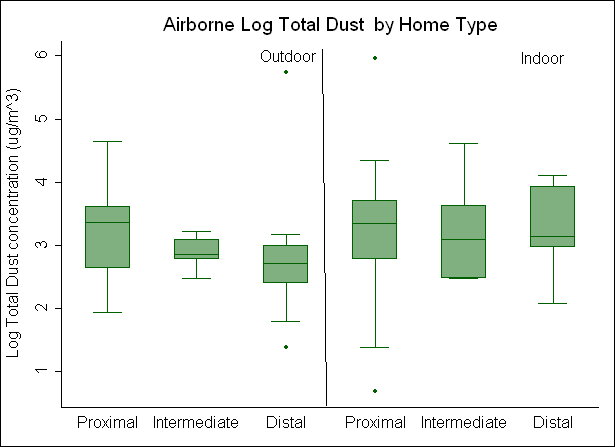
**Comparison of outdoor and indoor airborne concentrations of total dust between proximal, intermediate and distal home environments**.

Outdoor sampling results by distance classification are presented in Table 3. Outdoor results for airborne Bos d 2 showed the highest concentrations outside of proximal homes and lowest concentrations outside distal homes suggesting a concentration gradient. Median outdoor airborne cow allergen concentrations were 0.66 μg/m^3^, 0.17 μg/m^3^, and 0.01 μg/m^3 ^for proximal, intermediate and distal homes, respectively. Box plots showing log concentrations of outdoor Bos d 2 by distance group are presented in Figure 2. Ammonia concentrations (Figure 3) demonstrated a similar gradient (median 8.7 ppb proximal, 1.3 ppb intermediate, 1.1 ppb distal), with concentrations outside proximal homes significantly greater than concentrations outside homes classified as intermediate and distal. Following the same pattern, TD concentrations, presented in Figure 4, are significantly greater in outdoor environments of proximal (median: 29 μg/m^3^) compared to distal homes (median: 15 μg/m^3^), but not significantly greater than intermediate homes (median: 18 μg/m^3^). Median outdoor Bos d 2, ammonia, and TD were 60, eight and two times higher respectively, in the proximal as compared to the distal.

**Table 3:** Outdoor Air Samples - Proximal, Intermediate and Distal Homes

Analyte	Home Type	n(< LOD)	mean	sd	median	IQR	max		*p *value*
Bos d 2 μg/m^3^	Proximal Intermediate Distal	19 (0) 6 (0) 12 (3)	0.77 0.28 0.028	0.56 0.44 0.026	0.66 0.17 0.011	0.79 0.1 0.03	1.87 0.292 0.096	P vs D P vs I D vs I	< 0.01 < 0.01 < 0.01

NH_3 ppb_	Proximal Intermediate Distal	19 (0) 7 (1) 13 (6)	9.4 1.9 1.0	5.8 2.0 0.6	8.7 1.3 1.1	6.3 1.1 0.8	28.0 6.4 2.5	P vs D P vs I D vs I	< 0.01 < 0.01 0.24

TD μg/m^3^	Proximal Intermediate Distal	19 (0) 6 (0) 13 (0)	33 18 37	24 5 82	29 18 15	23 6 9	104 25 310	P vs D P vs I D vs I	0.02 0.09 0.38

*Kruskal-Wallis

P = proximal, D = distal and I = intermediate home types.

### Indoor Air

Indoor air results classified by distance are summarized in Table 4. Median indoor Bos d 2 concentrations were significantly greater in proximal, (0.12 μg/m^3^), compared to intermediate, (0.01 μg/m^3^), and distal (0.01 μg/m^3^) homes (Figure 2). Ammonia concentrations inside proximal homes (12 ppb) were greater than intermediate (4.9 ppb) and distal homes (5.7 ppb) (Figure 3). Differences between proximal and distal, and proximal and intermediate were statistically significant, while differences between intermediate and distal were not. Median indoor airborne Bos d 2 and ammonia concentrations were approximately 10 and two times higher respectively, in proximal as compared to distal homes. Indoor TD concentrations were similar for all three home classifications (Figure 4). No significant difference was seen between indoor concentrations for intermediate and distal homes for any airborne contaminant.

**Table 4:** Indoor Air Samples - Proximal, Intermediate and Distal Homes

Analyte	Home Type	n(< LOD)	mean	sd	median	IQR	max		*p *value*
Bos d 2 μg/m^3^	Proximal Intermediate Distal	16 (1) 5 (1) 12 (6)	0.20 0.04 0.010	0.27 0.01 0.006	0.12 0.01 0.011	0.24 0.29 0.001	0.97 0.12 0.029	P vs D P vs I D vs I	< 0.01 0.05 0.15

NH_3 ppb_	Proximal Intermediate Distal	20 (0) 7 (0) 13 (0)	15.7 10.0 5.9	15.1 9.9 3.3	12 4.9 5.7	12.6 20.5 4.4	56.0 25.5 12	P vs D P vs I D vs I	0.01 0.28 0.66

TD μg/m^3^	Proximal Intermediate Distal	18 (0) 7 (0) 12 (0)	47 34 33	86 31 19	29 22 23	25 26 32	385 100 61	P vs D P vs I D vs I	0.70 0.90 0.67

*Kruskal-Wallis

P = proximal, D = distal and I = intermediate home type.

### Indoor and Outdoor Concentrations

Indoor and outdoor concentrations of airborne contaminants were also compared within home type. Median outdoor airborne concentrations of Bos d 2 were significantly higher at proximal and intermediate homes (0.66 vs. 0.17 μg/m^3^) compared to indoor concentrations (0.12 vs. 0.01 μg/m^3^). This difference was not noted in distal homes, since concentrations were much lower and often below the limit of detection. Indoor concentrations of ammonia were higher than outdoor concentrations in all three groups; however, no significant difference was observed between indoor and outdoor ammonia concentrations in proximal homes (12 vs. 9 ppb). For intermediate and distal homes, a significant difference was found between indoor and outdoor ammonia concentrations with indoor levels being greater, 5 vs. 1 ppb and 6 vs. 1 ppb respectively. TD was significantly higher indoors compared to outdoors in distal homes, 23 vs. 15 μg/m^3^, while there were no significant differences in proximal or intermediate homes, 29 vs. 29 μg/m^3 ^and 22 vs. 18 μg/m^3 ^respectively.

As non-smoking participants were selected for this study, nicotine sampling was used to determine compliance with this criterion and to evaluate potential discrepancies in observed PM levels. Only one proximal home had measurable airborne nicotine. As a result indoor measurements of TD for this home were not included in any of the analyses presented above.

## Discussion

In this study we showed that outdoor PM, ammonia and cow allergen concentrations displayed a gradient with the highest concentrations inside and outside of homes closest to dairies (within a ¼ mile, 0.4 km) and the lowest concentrations outside of homes farthest from dairies (greater than three miles, 4.8 km). While many pollutants associated with dairy facilities can have multiple sources, complicating source attribution, cow allergen was selected because it is uniquely associated with the presence of cows. Homes with resident cows or homes where there was an individual that worked with cows were excluded to minimize the influence of occupational exposures on indoor environments. As a result, the presence of cow allergens inside and outside of homes is most likely attributed to emissions from dairy facilities. Similarly the ammonia concentration gradient implicates dairy operations as they are the only known major ambient source of ammonia in the study area. While PM can have multiple sources, our data also implicate dairies as a source of elevated PM concentrations outside households within a ¼ mile (0.4 km) of the facilities as compared to homes farther away.

Another key finding is that indoor pollutant concentrations also exhibit a concentration gradient with distance from dairy operations. In addition, indoor and outdoor concentrations of ammonia at homes within a ¼ mile (0.4 km) are indistinguishable, while the difference in indoor and outdoor ammonia concentrations in intermediate and distal homes is significantly different, with indoor being higher. These results indicate that being inside homes close to dairy operations provides little or no protection.

While the public health relevance of chronic exposure to cow allergen has yet to be established, occupational studies of health effects related to Bos d 2 allergen and sensitization in exposed dairy workers suggests that concentrations do not need to be extremely high for sensitization to occur [33,39-41]. For residents adjacent to dairy operations, exposure to cow allergen may have important health implications because sensitized individuals can experience allergic symptoms. Allergen exposure among sensitized individuals with asthma may serve as a trigger of respiratory symptoms and have been linked to the increased need for medication use and health care services [42,43]. To the extent that cow allergen can serve as a marker for biological components of dairy-related PM it is reasonable to conclude that other components not measured in this study, such as chemical agents, endotoxin, antibiotics, and/or microorganisms, are likely also to be elevated in the air outside and inside homes closest to dairy facilities.

In the case of ammonia, our results are consistent with Atkins who found that people and pets can be important contributors to indoor concentrations of ammonia [44]. In distal and intermediate homes, indoor ammonia concentrations were significantly greater than those measured outdoors. However, for proximal homes, indoor ammonia concentrations were only slightly and non-significantly higher than outdoors. These results suggest that ammonia penetration from outdoors is a significant contributor to indoor ammonia concentrations for homes close to dairy operations. The five-day average proximal outdoor concentrations measured in our study are similar to other studies that have measured ammonia using comparable methods around swine facilities [15,36,45]. While we did not measure health outcomes or quality of life indicators, other studies of communities located within two to three miles (3.2 to 4.8 km) of an IFAP facility [12,15,16], found that odors and ammonia can contribute to poor quality of life even at ammonia concentrations currently considered not to be a risk to health. Our findings demonstrate that exposure to ammonia increases as distance to the facility decreases. This suggests that quality of life may be even further diminished as the indoor environment in proximate homes provides no refuge from this gas.

Agricultural dusts, which are primarily composed of particles in the larger size fractions, can have profound effects on local populations that are chronically exposed [46,47]. It has been shown that larger particles can be carriers of important biological agents [23,48,49]. In addition, inhalable particles have been associated with increased asthma, sinusitis, rhinitis and upper airway diseases in agricultural workers [11,50-55]. Several studies have found evidence that indoor coarse particles may be associated with increased incidence of asthma symptoms in urban populations [56-59]. Population based studies, which have evaluated PM concentrations in ambient environments, also support the importance of the size and composition of ambient PM to morbidity and mortality [60-62]. These studies have been conducted primarily in urban environments and currently there is limited data about the influence of PM on morbidity and mortality in rural and agricultural environments.

There are a number of limitations to this study. Since the data collection was cross-sectional, trends over time or across seasons cannot be evaluated. The sample size, while sufficiently large to address the issue associated with proximity, is too small to provide a comprehensive assessment of the ranges of exposures associated with living close to industrial dairy operations. In addition, integrated sampling methods cannot evaluate important short-term within week and within day variability, which may be subject to exceptionally high concentrations. This is particularly important for ammonia where elevated short-term exposures can result in significant irritation and health effects.

Airborne PM samples collected in this study utilized a 37-mm close-faced sampling cassette. This sampler was used to estimate airborne PM concentrations and to assess airborne cow allergen concentrations. This sampler has been shown to underestimate the inhalable fraction of airborne PM in general and in swine barns in particular it has a > 80% collection efficiency for particles up to approximately 10 μm in diameter [63,64]. The degree to which the TD sampler underestimates inhalable dust exposure will depend on the particle size distribution, face-velocity, and wind speed [64]. In swine barns the TD sampler underestimated inhalable fraction by about 14% [63]. If a significant fraction of allergen-containing particles are greater than 10 μm in aerodynamic diameter, TD sampling will underestimate cow allergen concentrations

Information on home cleanliness, use of cow manure in home gardens, wind direction, orientation to facility, specific farming processes used, number of facilities, facility size, and actual number of animals should be collected in future studies to allow for a better assessment of concentration distributions and source attribution.

## Conclusions

This is one of the first studies to provide evidence of a gradient of pollutant concentrations by distance of homes to industrial scale dairy operations. Concentrations of Bos d 2, ammonia, and PM were significantly higher for homes within a ¼ mile (0.4 km) of a facility or associated sprayfield compared with homes more than three miles (4.8 km) away. These findings reinforce community concerns of exposure and substantiate the need for larger, well-designed environmental exposure and health effects studies to determine the influence of these facilities and their contaminants on health in adjacent communities. In addition these results have important implications for dairy facility siting policy decisions, nutrient management plans, and zoning of IFAP when located close to communities. Furthermore, these results highlight the need to consider developing IFAP emissions standards and air pollution regulations in order to protect public health.

## List of abbreviations

Bos d 2: a cow allergen; FRM: Federal Reference Method; GIS: Geographic Information System; IFAP: Industrial Food-Animal Production; JHSPH: Johns Hopkins Bloomberg School of Public Health; LOD: Limit of detection; NH_3_: ammonia; PM: particulate matter; PM_2.5_: particulate matter less than or equal to 2.5 microns in aerodynamic diameter; PM_10-2.5_: PM less than10 but greater than 2.5 microns in aerodynamic diameter; PM_10_: PM less than or equal to10 microns in aerodynamic diameter; TD: total dust; USDA: United States Department of Agriculture; US EPA: United State Environmental Protection Agency.

## Competing financial interest declaration

The authors declare that they have no competing interests.

## Authors' contributions

DLW obtained funding for the work. In addition she designed, executed, and conducted data analysis for the study. She was also the lead author. PNB obtained funding for the work, contributed to the study design, result interpretation and contributed the drafting of the article. MCM contributed to the design of the study, result interpretation and provided substantial revisions to the document. GBD contributed to the editing and revision of the document. SM helped with the conceptual design, result interpretation and revisions to the document. ASG contributed to the study design, result interpretation and contributed substantially to the drafting of the article. All authors read and approved the final manuscript.
